# The *BDNF* val-66-met Polymorphism Affects Neuronal Morphology and Synaptic Transmission in Cultured Hippocampal Neurons from Rett Syndrome Mice

**DOI:** 10.3389/fncel.2017.00203

**Published:** 2017-07-13

**Authors:** Xin Xu, Jordi Garcia, Rachel Ewalt, Shelly Nason, Lucas Pozzo-Miller

**Affiliations:** Department of Neurobiology, Civitan International Research Center, University of Alabama at Birmingham Birmingham, AL, United States

**Keywords:** BDNF val66met, Rett syndrome, dendritic branching, dendritic spines, synaptic transmission

## Abstract

Brain-derived neurotrophic factor (*Bdnf*) has been implicated in several neurological disorders including Rett syndrome (RTT), an X-linked neurodevelopmental disorder caused by loss-of-function mutations in the transcriptional modulator methyl-CpG-binding protein 2 *(MECP2)*. The human *BDNF* gene has a single nucleotide polymorphism (SNP)—a methionine (met) substitution for valine (val) at codon 66—that affects BDNF’s trafficking and activity-dependent release and results in cognitive dysfunction. Humans that are carriers of the met-BDNF allele have subclinical memory deficits and reduced hippocampal volume and activation. It is still unclear whether this *BDNF* SNP affects the clinical outcome of RTT individuals. To evaluate whether this BDNF SNP contributes to RTT pathophysiology, we examined the consequences of expression of either val-BDNF or met-BDNF on dendrite and dendritic spine morphology, and synaptic function in cultured hippocampal neurons from wildtype (WT) and *Mecp2* knockout (KO) mice. Our findings revealed that met-BDNF does not increase dendritic growth and branching, dendritic spine density and individual spine volume, and the number of excitatory synapses in WT neurons, as val-BDNF does. Furthermore, met-BDNF reduces dendritic complexity, dendritic spine volume and quantal excitatory synaptic transmission in *Mecp2* KO neurons. These results suggest that the val-BDNF variant contributes to RTT pathophysiology, and that BDNF-based therapies should take into consideration the *BDNF* genotype of the RTT individuals.

## Significance Statement

The neuroprotective effects of BDNF have been demonstrated in various animal models of neurological and psychiatric disorders. BDNF dysfunction has been implicated in pathophysiological mechanisms of Rett syndrome, the most common intellectual disability in women after Down syndrome (1:10,000 incidence). The *BDNF* val66met polymorphism is carried by approximately 30% of people worldwide and has been associated with cognitive deficits. Whether this *BDNF* single nucleotide polymorphism (SNP) contributes to RTT and how it affects the clinical outcome of RTT individuals remain unclear. Our findings help to understand the impact of val66met polymorphism on neuronal morphology and excitatory synaptic transmission in Rett mice, extend the current knowledge regarding BDNF’s role in RTT pathophysiology, providing pre-clinical evidence for BDNF-based therapies for RTT individuals.

## Introduction

Brain-derived neurotrophic factor (*Bdnf*) has been implicated in several neurological disorders due to its widespread function in neuronal development, plasticity, differentiation and survival (Poo, 2001; Fahnestock et al., 2002; Gines et al., 2010; Hartmann et al., 2012). The main function of BDNF in the adult brain is to regulate synaptic strength, promote synaptic growth and participate in plasticity-related processes underlying learning and memory (Tyler et al., 2002; Yamada et al., 2002; Lu, 2003a; Lu et al., 2013). A reduction of BDNF levels can cause impaired synaptic transmission and plasticity, reduced number of synapses and deficits in learning and memory in various pathological conditions (Mu et al., 1999; Durany et al., 2000; Ferrer et al., 2000; Lu et al., 2013).

The human *BDNF* gene has a SNP—a methionine (met) substitution for valine (val) at codon 66—that affects BDNF trafficking and activity-dependent release (Egan et al., 2003). This SNP is associated with a variety of neuropsychiatric disorders and cognitive dysfunction (Momose et al., 2002; Neves-Pereira et al., 2002; Ventriglia et al., 2002; Egan et al., 2003; Sen et al., 2003; Lu et al., 2013). BDNF promotes synapse formation and refinement by regulating axonal and dendritic branching and growth (Cohen-Cory and Fraser, 1995; McAllister et al., 1996; Lu et al., 2013). Decreased BDNF release may alter neuronal morphology, leading to changes in brain volume. Indeed, several studies have reported that humans and rodents carrying the met BDNF allele display smaller hippocampal volumes and exhibit profound deficits in hippocampal-dependent memory tasks, suggestive of reduced neuroplasticity (Egan et al., 2003; Hariri et al., 2003; Pezawas et al., 2004; Szeszko et al., 2005; Chen et al., 2006; Ninan et al., 2010; Bath et al., 2012; Baj et al., 2013).

BDNF has been implicated in Rett syndrome (RTT), an X-linked neurological disorder caused by loss-of-function mutations in the transcriptional modulator methyl-CpG-binding protein 2 (*MECP2*; Amir et al., 1999; Percy and Lane, 2005; Bienvenu and Chelly, 2006; Chahrour and Zoghbi, 2007). MeCP2 binds to the *Bdnf* promoter and directly regulates *Bdnf* expression in an activity-dependent manner (Chen et al., 2003; Martinowich et al., 2003; Zhou et al., 2006). *Bdnf* mRNA and protein levels are lower in MeCP2-deficient models and RTT individuals (Chang et al., 2006; Wang et al., 2006; Ogier et al., 2007; Li et al., 2012), and its overexpression rescues cellular and behavioral deficits (Chang et al., 2006; Chahrour and Zoghbi, 2007; Larimore et al., 2009). Dysfunctional BDNF signaling has been demonstrated in several pathophysiological mechanisms of RTT disease progression (Katz, 2014; Li and Pozzo-Miller, 2014), but the contribution of the *BDNF* val66met SNP to RTT symptoms remains unclear: one study reported that the met *BDNF* allele is protective for seizure onset in RTT individuals (Nectoux et al., 2008), while another described that it leads to earlier seizure onset (Zeev et al., 2009). Therefore, it is highly relevant to characterize the role of this *BDNF* SNP in synaptic and cellular features of *Mecp2* deficient neurons from RTT mice. Our findings revealed that while met-BDNF does not promote dendritic growth and excitatory synapse formation in wildtype (WT) neurons, it actually reduces dendritic complexity, dendritic spine volume and quantal excitatory synaptic transmission in *Mecp2* knockout (KO) neurons.

## Materials and Methods

### Animals

Breeding pairs of mice lacking exon 3 of the X chromosome-linked *Mecp2* gene (B6.Cg-*Mecp2*^tm1.1Jae^, “Jaenisch” strain in a pure C57BL/6 background; Chen et al., 2001) were purchased from the Mutant Mouse Regional Resource Center at the University of California, Davis. A colony was established at the University of Alabama at Birmingham (UAB) by mating WT males with heterozygous *Mecp2*^tm1.1Jae^ mutant females, as recommended by the supplier. Genotyping was performed by PCR of DNA sample from tail clips. Hemizygous *Mecp2*^tm1.1Jae^ mutant males (called KOs) are healthy until 5–6 weeks of age, when they begin to show RTT-like symptoms, such as hypoactivity, hind limb clasping, reflex impairments and irregular breathing (Chen et al., 2001). Animals were handled and housed according to the Committee on Laboratory Animal Resources of the National Institutes of Health; all experimental protocols were reviewed annually and approved by the Institutional Animals Care and Use Committee of the UAB.

### Primary Culture of Hippocampal Neurons and Transfections

Both hippocampi were dissected from anesthetized postnatal day 0 or 1 (P0–1) male* Mecp2* KO mice and WT littermates, and dissociated in papain (20 U/ml) plus DNase I (Worthington) for 20–30 min at 37°C, as described (Amaral and Pozzo-Miller, 2007). The tissue was then triturated to obtain a single-cell suspension, and the cells were plated at a density of 40,000 cells/cm^2^ on 18 mm poly-L-lysine/laminin coated glass coverslips, and immersed in Neurobasal medium (Life technologies) supplemented with 2% B27 (Life technologies) and 0.5 mM glutamine (Life technologies). Neurons were grown in 37°C, 5% CO_2_, 90% relative humidity incubators (Thermo-Forma), with half of the fresh medium changed every 3–4 days. After 7–8 days *in vitro* (DIV), cDNA plasmids encoding either human val-BDNF or met-BDNF (tagged with green fluorescent protein GFP for their localization; 1.6 μg DNA; a gift from Dr. Masami Kojima) were transfected alone or in combination with soluble GFP (for imaging neuronal morphology) using Lipofectamine 2000 (Life technologies) according to the protocol of the manufacturer.

### Immunocytochemistry

Neurons were fixed 48 h after transfection with 4% (wt/vol) paraformaldehyde/sucrose for 10 min, and incubated in 0.25% (vol/vol) Triton X-100 for 10 min, then washed with PBS. After blocking with 10% (vol/vol) goat serum in PBS, cells were incubated with anti-GFP antibody (1:2000, ab290, rabbit pAb, Abcam) overnight at 4°C, rinsed in PBS, and incubated with Alexa Fluor 488 secondary antibody (1:500, Life Technologies) for 1 h; coverslips were then mounted with Vectashield medium (Vector Laboratories). Images were acquired in a laser-scanning confocal microscope using a solid-state 488 nm laser for excitation, and a 60× 1.4 NA oil immersion lens, and standard FITC dichroic and emission filters (FluoView-300, Olympus; Center Valley, PA, USA). Excitatory synapses were identified with antibodies against glutamate AMPA receptors (GluA1; 1:100, MAB2263, mouse mAb, Millipore) and vesicular glutamate transporter (VGLUT1; 1:300, AB5905, guinea pig pAb, Millipore), and imaged in a confocal microscope (Zeiss LSM510) using a 63× 1.4 NA oil-immersion objective.

### Electrophysiology

Coverslips with hippocampal 9–11 DIV neurons were continuously perfused with artificial CSF (aCSF) containing (in mM): 130 NaCl, 5.4 KCl, 2 CaCl_2_, 1.2 MgCl_2_, 20 HEPES, 15 glucose; pH 7.4. Miniature excitatory postsynaptic currents (mEPSC) were recorded in the whole-cell configuration from pyramidal shaped neurons voltage clamped at −70 mV using an Axopatch 200B amplifier (Molecular Devices). The intracellular solution contained (in mM): 120 Cs-gluconate, 17.5 CsCl, 10 Na-HEPES, 4 Mg-ATP, 0.4 Na-GTP, 10 Na_2_-creatine phosphate, 0.2 Na-EGTA, pH 7.4, 300 mOsm. To pharmacologically isolate mEPSCs, the aCSF contained 0.5 μM TTX, 50 μM D-AP5 and 50 μM picrotoxin (Sigma). Whole-cell currents were digitized at 10 kHz and filtered at 2 kHz. Cells with series resistance >20 MΩ or that changed by ≥20% during the recording are excluded. mEPSCs were detected and analyzed using the MiniAnalysis program (Synaptosoft).

### Image Analysis

The morphology of neurons was analyzed with the Filament Tracing and Surface Rendering modules of Imaris software (Bitplane). The fraction of cell body or dendrites filled with either val-BDNF-GFP or met-BDNF-GFP relative to the total territory filled with either val-BDNF-GFP or met-BDNF-GFP was calculated using ImageJ using the same threshold for fluorescence intensity. To trace spines, a region of interest (ROI) was selected and a new filament was created using the Autopath mode, as previously described (Swanger et al., 2011). The *minimum dendrite end diameter* was set at 0.75 μm, and automatic thresholds were used for dendrite surface rendering. The *maximum spine length* was set at 5 μm. Protrusions longer than 5 μm were rarely observed in neurons at DIV 10 and were not considered as spines. Spines were manually counted and spine density was calculated by quantifying the number of spines per dendritic segment, and normalized to 10 μm of dendrite length. Sholl analysis and branch order analyses were performed using NeuronStudio software (Wearne et al., 2005). The density of excitatory synapses was determined by the number of VGLUT1/GluA1 co-localized puncta per length of GFP-positive dendrite, and normalized to 10 μm of dendrite length. Three randomly selected segments of primary or secondary dendrites (30–40 μm for each segment) were analyzed for spine density and synaptic density; these dendritic segments were located at least one soma diameter away from the soma, and were void of crossing dendrites and axons from other neurons.

### Statistical Analyses

Data were presented as mean ± standard error of the mean (SEM), and were compared using unpaired Student’s *t*-test, one-way ANOVA, or Kolmogorov–Smirnov (K–S) test using Prism software (GraphPad Software, San Diego, CA, USA). Statistical Power was calculated using G*Power (Faul et al., 2007); *p* < 0.05 was considered significant.

## Results

### BDNF-GFP Trafficking Is Impaired in Hippocampal Neurons Expressing met-BDNF

**Figure 1:**
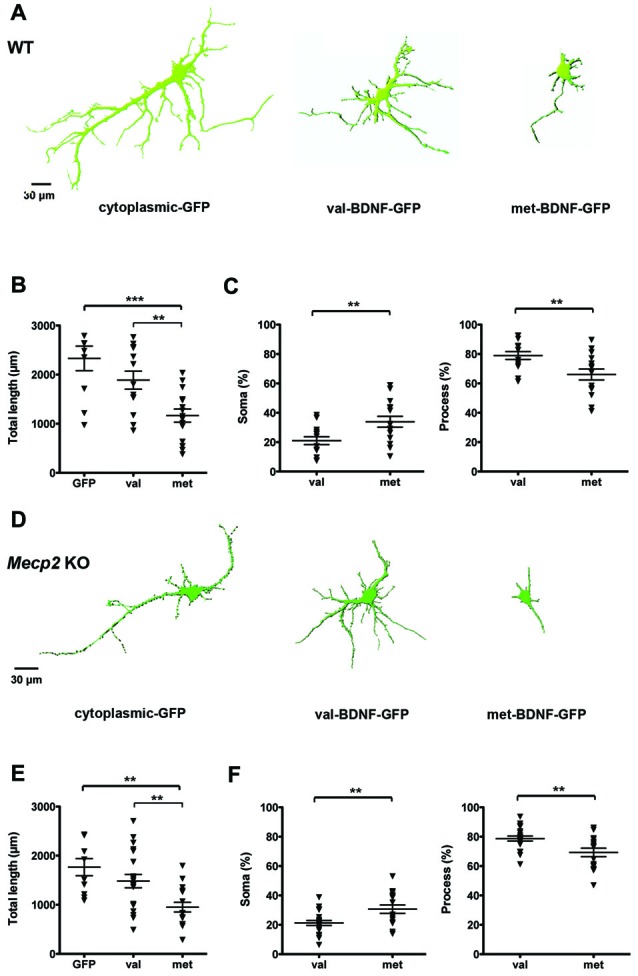
Brain-derived neurotrophic factor (BDNF)-GFP trafficking is impaired in wildtype (WT) and *methyl-CpGbinding protein 2 (Mecp2)* knockout (KO) hippocampal neurons expressing met-BDNF. **(A)** Representative surface rendering of 3D reconstructions of WT hippocampal neurons expressing soluble GFP alone (neuronal morphology), val-BDNF-GFP alone, or met-BDNF-GFP alone. Note that the GFP tags of val-BDNF and met-BDNF show their cellular distribution and not the full morphology of the neurons, which is shown by soluble GFP. **(B)** Total length of dendrites containing soluble GFP, val-BDNF-GFP, or met-BDNF-GFP. **(C)** Fraction of either val-BDNF or met-BDNF present in somata and dendrites, over the total area occupied by BDNF-GFP (soma plus dendrites). **(D)** Representative surface rendering of 3D reconstructions of *Mecp2* KO hippocampal neurons expressing soluble GFP (complete neuronal morphology) alone, val-BDNF-GFP alone, or met-BDNF-GFP alone. **(E)** Total length of dendrites containing soluble GFP, val-BDNF-GFP, or met-BDNF-GFP. **(F)** Fraction of either val-BDNF or met-BDNF present in somata and dendrites, over the total area occupied by BDNF-GFP (soma plus dendrites). **p < 0.01, ***p < 0.001.

We first characterized the distribution of GFP-tagged BDNF in cultured hippocampal neurons from WT mice that were transfected with either val-BDNF or met-BDNF. Forty-eight hours after transfection with cDNA plasmids, BDNF-GFP filled most of the dendritic tree of val-BDNF-expressing neurons, throughout their distal secondary and tertiary dendrites, with a total length comparable to neurons expressing only cytoplasmic GFP (Figures 1A,B; GFP = 2332 ± 250.1 μm, *n* = 10 neurons; val-BDNF = 1888 ± 184.1 μm, *n* = 14; *p* = 0.0791). However, BDNF-GFP was restricted to the cell body and most proximal regions of primary dendrite of neurons expressing met-BDNF. The dendritic length filled with BDNF-GFP in met-BDNF-expressing neurons was significantly smaller than that in val-BDNF-expressing neurons (Figures 1A,B; met-BDNF = 1166 ± 132.7 μm, *n* = 15; *p* = 0.0016). Consistently, met-BDNF-expressing neurons showed a larger fraction of soma and a smaller fraction of dendrites filled with BDNF-GFP compared to val-BDNF-expressing neurons (Figure 1C; val-BDNF soma = 21.02 ± 2.68%; met-BDNF soma = 33.91 ± 3.72%; *p* = 0.0049). These results are consistent with previous studies describing impaired met-BDNF trafficking into distal dendrites (Egan et al., 2003; Chen et al., 2004, 2006). Such restricted distribution of BDNF-GFP was also observed in met-BDNF-expressing hippocampal neurons from *Mecp2* KO mice (Figures 1D–F; GFP = 1766 ± 175.4 μm, *n* = 10 neurons; val-BDNF = 1483 ± 135.6 μm, *n* = 20; *p* = 0.1130 GFP vs. val; met-BDNF = 952 ± 98.6 μm, *n* = 17; *p* = 0.0021 val vs. met; val-BDNF soma = 21.27 ± 1.73%; met-BDNF soma = 30.68 ± 2.88%; *p* = 0.0032).

### Opposite to val-BDNF, met-BDNF Reduces Dendritic Length and Branching in Wildtype and *Mecp2* Knockout Neurons

**Figure 2:**
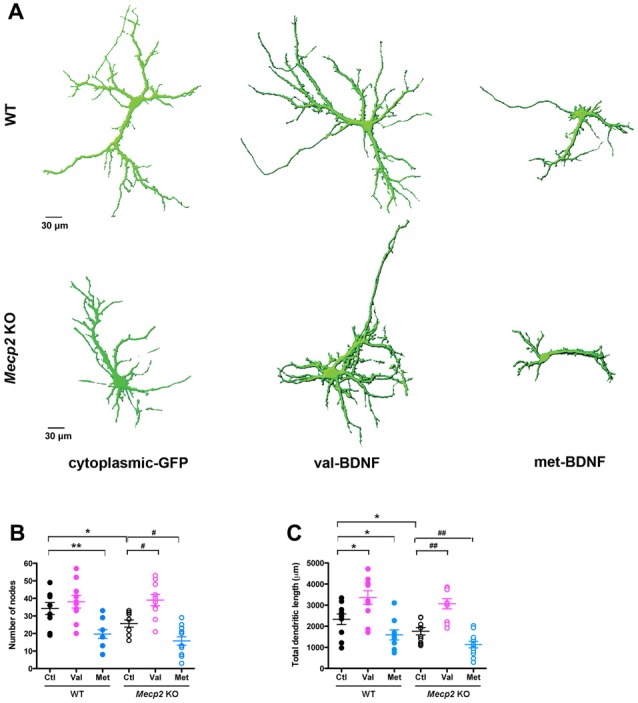
val-BDNF increases, and met-BDNF reduces dendritic length and branching in both *Mecp2* KO and WT neurons, without affecting axonal morphology.** (A)** Representative 3D reconstructions of WT and *Mecp2* KO hippocampal neurons expressing either soluble GFP alone, val-BDNF and soluble GFP, or met-BDNF and soluble GFP. Note that these are images from anti-GFP immunofluorescence labeling, and they reveal the full morphology of the neurons (the GFP tags of val-BDNF and met-BDNF are masked by soluble GFP, which fills the entire cell). **(B)** Number of dendritic branch points (nodes). **(C)** Total dendritic length, identified as MAP2(+) processes. *p < 0.05, **p < 0.01, compared to WT; ^#^*p* < 0.05, ^##^*p* < 0.01 compared to *Mecp2* KO.

BDNF promotes synapse formation and refinement by regulating axonal and dendritic growth and branching (Cohen-Cory and Fraser, 1995; McAllister et al., 1996; Lu et al., 2013). A reduction in BDNF secretion may affect dendritic and axonal growth. To examine the consequences of the *BDNF* SNP on dendritic morphology, either val-BDNF or met-BDNF were co-transfected with soluble GFP to fill the entire neuron. In WT neurons, expression of val-BDNF increased dendritic length (Figures 2A,C; WT Ctl = 2332 ± 250.1 μm, *n* = 10 neurons; WT val-BDNF = 3360 ± 321.5 μm, *n* = 10; *p* = 0.0106), but not the branching (Figures 2A,B; WT Ctl = 34.2 ± 3.4 nodes; WT val-BDNF = 38.1 ± 3.6 nodes; *p* = 0.2226). On the other hand, expression of met-BDNF significantly reduced dendritic length (Figures 2A,C; WT met-BDNF = 1593 ± 240.2 μm, *n* = 10; *p* = 0.0245) and branching (Figures 2A,B; WT met = 19.7 ± 2.5 nodes; *p* = 0.0018).

Of relevance to RTT and consistent with *in vivo* and *in vitro* observations (Armstrong et al., 1995; Fukuda et al., 2005; Schüle et al., 2008; Belichenko et al., 2009; Baj et al., 2014), *Mecp2* KO neurons showed shorter dendrites with fewer branch points than WT neurons (Figures 2A–C; KO Ctl = 1766 ± 175.4 μm, *n* = 10 neurons; *p* = 0.0401; KO Ctl = 25.7 ± 2.0 nodes; *p* = 0.0237). As we showed after shRNA-mediated MeCP2 knockdown (Larimore et al., 2009), expression of val-BDNF increased dendritic length and branching in *Mecp2* KO neurons (Figures 2A–C; KO val-BDNF = 3063 ± 240.4 μm, *n* = 10; *p* = 0.0015; KO val-BDNF = 39.0 ± 3.2 nodes; *p* = 0.013). On the other hand, expression of met-BDNF significantly reduced dendritic length and branching in *Mecp2* KO neurons (Figures 2A–C; KO met-BDNF = 1130 ± 153.8 μm, *n* = 12; *p* = 0.0063; KO met-BDNF = 15.8 ± 2.4 nodes; *p* = 0.036).

**Figure 3:**
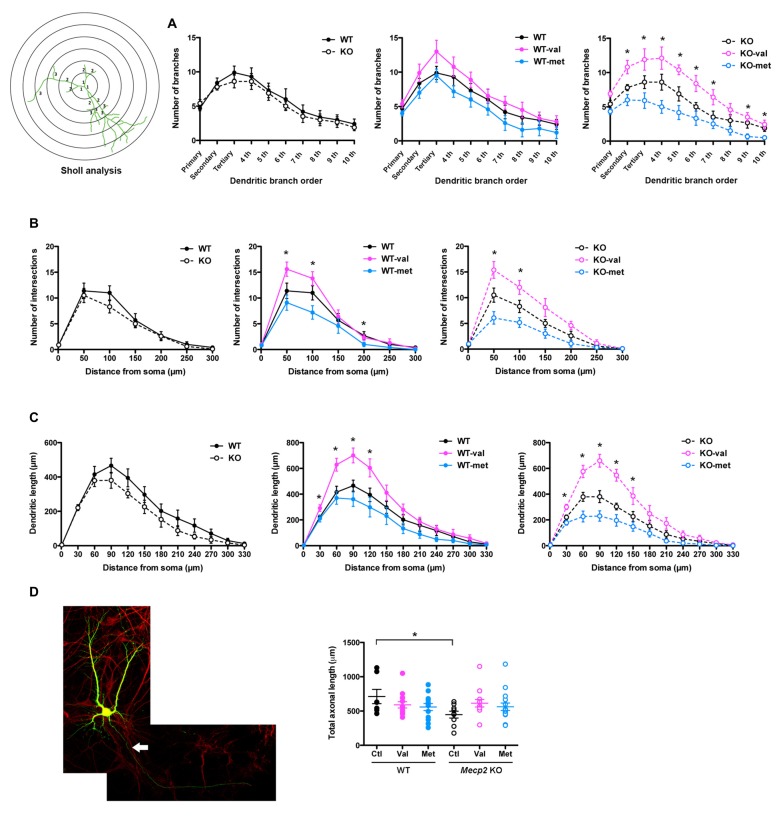
val-BDNF increases, and met-BDNF reduces dendritic complexity. **(A)** Number of dendritic branches of each branch order (primary, secondary, tertiary, 4th, etc.; see branch orders in a representative cell in **(B)**. **(B)** Number of dendritic intersections as a function of distance from the soma. Sholl analysis using concentric circles starting at the soma in 50 μm increments (anti-GFP immunofluorescence). **(C)** Total dendritic length in each concentric segment, as a function of distance from the soma. **(D)** Left: representative images of MAP2 staining after neuronal transfection. The axon of neuron is shown by arrow. Right: total axonal length, identified as the only MAP2(−) process in each cell. *p < 0.05 between genotypes and transfections.

Sholl analysis of dendrites provided additional details of the effects of val-BDNF expression on dendritic complexity (Figure 3). Expression of neither val-BDNF nor met-BDNF affected the number of dendritic branches in WT neurons. On the other hand, expression of val-BDNF increased branch number, while met-BDNF expression decreased branch number in *Mecp2* KO neurons (Figure 3A; secondary *p* = 0.0198; tertiary *p* = 0.0450; 4th order *p* = 0.0198; 5th order *p* = 0.0170; 6th order *p* = 0.0267; 7th order *p* = 0.0279; 9th order *p* = 0.0128; 10th order *p* = 0.0073). Regarding dendritic length within Sholl concentric circles, val-BDNF expression increased it in both WT and *Mecp2* KO neurons, while met-BDNF expression reduced it in both genotypes. These effects were statistically significant at a 30–120 μm radius from the cell body (Figure 3B; WT *p* = 0.0175 at 30 μm, *p* = 0.0029 at 60 μm, *p* = 0.0021 at 90 μm, *p* = 0.0139 at 120 μm; *Mecp2* *p* = 0.0046 at 30 μm, *p* = 0.0077 at 60 μm, *p* = 0.003 at 90 μm, *p* = 0.0033 at 120 μm).

Regarding axonal morphology, *Mecp2* KO neurons showed shorter axons than WT neurons (Figure 3C; WT Ctl = 712.2 ± 103.8 μm, *Mecp2* Ctl = 448.6 ± 50.1 μm; *p* = 0.0137), but neither val-BDNF nor met-BDNF expression affected axonal length in either WT or* Mecp2* KO neurons (Figure 3C; WT val-BDNF = 590.1 ± 45.1 μm, *p* = 0.1101; WT met-BDNF = 558.8 ± 50.9 μm, *p* = 0.0756; KO val-BDNF = 614.2 ± 54.4 μm, *p* = 0.0663; KO met-BDNF = 563.3 ± 53.9 μm; *p* = 0.0861).

### Different to val-BDNF, met-BDNF Fails to Increase Dendritic Spine Density and Volume in *Mecp2* Knockout and Wildtype Neurons

**Figure 4:**
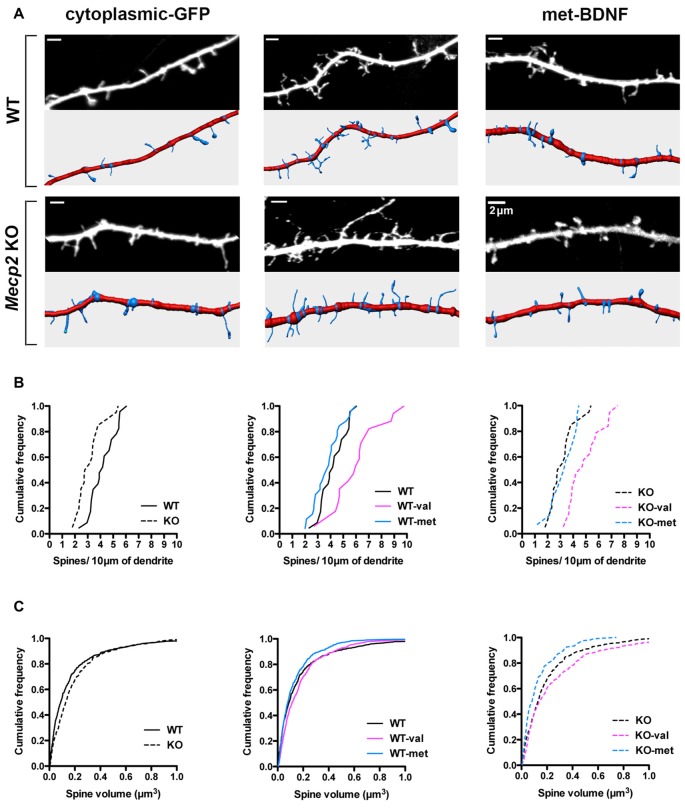
met-BDNF fails to increase dendritic spine density and volume like val-BDNF does in *Mecp2* KO and WT neurons.** (A)** Representative dendritic segments (top) and their reconstruction for automated spine detection and volume estimation (bottom) from WT and *Mecp2* KO neurons expressing either soluble GFP alone, val-BDNF/soluble GFP, or met-BDNF/soluble GFP (anti-GFP immunofluorescence). **(B)** Cumulative frequency of spine density per 10 μm of dendrite in WT and *Mecp2* KO neurons. **(C)** Cumulative frequency of spine volume in WT and *Mecp2* KO neurons.

Dendritic spines play a fundamental role in synaptic plasticity models of memory formation and storage, and their number and morphology are tightly correlated with synapse strength (Yuste and Bonhoeffer, 2001; Nimchinsky et al., 2002; Chapleau and Pozzo-Miller, 2008; Swanger et al., 2011). BDNF is a strong modulator of dendritic spine density and morphology in cortical and hippocampal pyramidal neurons (Horch and Katz, 2002; Tyler and Pozzo-Miller, 2003; Alonso et al., 2004; Chapleau et al., 2008). Of relevance to RTT and consistent with *in vivo* and *in vitro* observations (Belichenko et al., 1994; Chao et al., 2007; Chapleau et al., 2009a, 2012), cultured hippocampal neurons from *Mecp2* KO mice have a lower spine density than those from WT mice (Figures 4A,B; WT = 4.25 ± 0.28 spines/10 μm, total dendritic length 888.14 μm; *Mecp2* = 3.13 ± 0.3 spines/10 μm, total length 1377.83 μm; K-S test: *p* = 0.0054). However, the volume of individual spines is larger in *Mecp2* KO neurons than in WT neurons (Figures 4A,C; WT = 0.18 ± 0.02 μm^3^; *Mecp2* = 0.21 ± 0.01 μm^3^; *p* = 0.002), as observed in CA1 pyramidal neurons of symptomatic *Mecp2* KO mice and consistent with stronger excitatory synapses (Li et al., 2016).

Expression of val-BDNF increased dendritic spine density in both WT and *Mecp2* KO neurons: cumulative probability distributions showed a statistically significant shift towards higher density (Figures 4A,B; WT val-BDNF = 5.93 ± 0.54 spines/10 μm, total length 1079.72 μm; *p* = 0.0034; KO val-BDNF = 5.15 ± 0.38 spines/10 μm, total length 819.4 μm; *p* = 0.0001). In addition, val-BDNF expression increased the volume of individual spines in both WT and *Mecp2* KO neurons (Figures 4A,C; WT val-BDNF = 0.2 ± 0.01 μm^3^; *p* = 0.0026; KO val-BDNF = 0.3 ± 0.02 μm^3^; *p* = 0.0146). On the other hand, met-BDNF expression did not increase dendritic spine density neither in WT nor *Mecp2* KO neurons (Figures 4A,B; WT met-BDNF = 3.99 ± 0.29 spines/10 μm, total length 1154.42 μm; *p* = 0.3117; KO met-BDNF = 3.13 ± 0.34 spines/10 μm, total length 535.55 μm; *p* = 0.7243). Intriguingly, met-BDNF decreased spine volumes in *Mecp2* KO neurons, without affecting the volume of spines in WT neurons (Figures 4A,C; WT met-BDNF = 0.13 ± 0.01 μm^3^; *p* = 0.3340; KO met-BDNF = 0.16 ± 0.01 μm^3^; *p* = 0.0006).

### Opposite to val-BDNF, met-BDNF Reduces the Number of Excitatory Synapses in Wildtype Neurons, and Fails to Promote Excitatory Synaptogenesis in *Mecp2* Knockout Neurons

**Figure 5:**
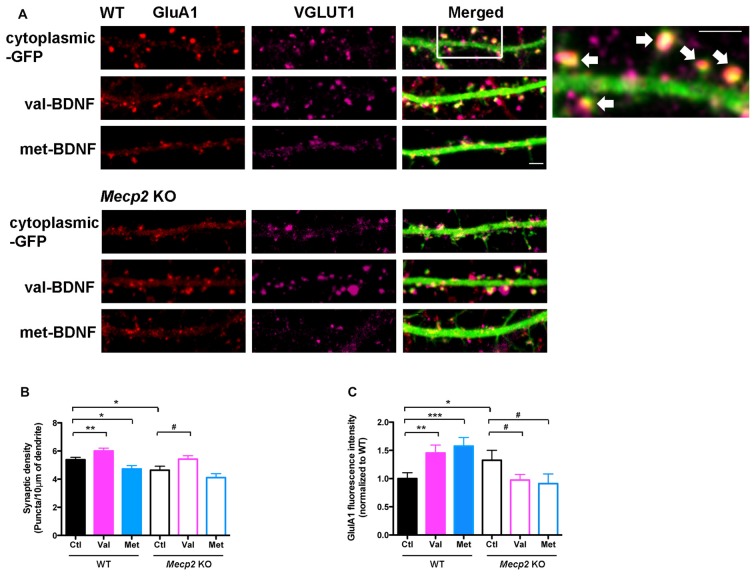
val-BDNF increases, and met-BDNF reduces the number of excitatory synapses. **(A)** Representative images of immunostaining for surface GluA1 (red) and VGLUT1 (purple) in WT and *Mecp2* KO neurons expressing either soluble GFP alone, val-BDNF/soluble GFP, or met-BDNF/soluble GFP; scale bar = 2 μm. Excitatory synapses are shown by arrows. **(B)** Numerical density of GluA1/VGLUT1 synaptic puncta in WT and *Mecp2* KO neurons. **(C)** Fluorescence intensity of GluA1 synaptic puncta in WT and *Mecp2* KO neurons. Fluorescence intensity was normalized to WT neurons. *p < 0.05, **p < 0.01, ***p < 0.001 compared to WT; ^#^*p* < 0.05 compared to *Mecp2* KO.

The formation of dendritic spines is critical and precedes the establishment of excitatory synapses (Chapleau and Pozzo-Miller, 2008; Giachero et al., 2013). The number of excitatory synapses, identified as VGLUT1-expressing presynaptic terminals apposed to dendritic spines of GFP-expressing neurons, and their surface levels of GluA1 were determined by triple color immunocytochemistry (Figure 5A). Confirming published observations (Chao et al., 2007; Baj et al., 2014), 7–8 DIV cultured hippocampal neurons from *Mecp2* KO mice have significantly fewer excitatory synapses than WT neurons (GluA1/VGLUT1 puncta; Figures 5A,B; WT Ctl = 5.38 ± 0.17 puncta/10 μm, *n* = 23 neurons; KO Ctl = 4.64 ± 0.28 puncta/10 μm, *n* = 12; *p* = 0.0100). However, excitatory synapses from *Mecp2* KO mice have significantly higher surface levels of GluA1 (Figures 5A,C; WT Ctl = 1 ± 0.1; KO Ctl = 1.33 ± 0.18; *p* = 0.0488), consistent with stronger excitatory synapses (Li et al., 2016).

Expression of val-BDNF significantly increased the number of excitatory synapses in both WT and *Mecp2* KO neurons (Figures 5A,B; WT val-BDNF = 6.01 ± 0.19 puncta/10 μm, *n* = 18; *p* = 0.0076; KO val-BDNF = 5.42 ± 0.24 puncta/10 μm, *n* = 20; *p* = 0.0235), whereas met-BDNF expression did not. In fact, expression of met-BDNF significantly decreased the number of excitatory synapses in WT neurons (Figures 5A,B; WT met-BDNF = 4.72 ± 0.12 puncta/10 μm, *n* = 15; *p* = 0.0107) to levels comparable to *Mecp2* KO neurons. On the other hand, met-BDNF expression did not affect the number of excitatory synapses in *Mecp2* KO neurons (met-BDNF = 4.11 ± 0.28 puncta/10 μm, *n* = 11; *p* = 0.0960). Intriguingly, expression of either val-BDNF or met-BDNF increased the surface levels of postsynaptic GluA1 subunits in WT neurons (Figures 5A,C; WT val-BDNF = 1.45 ± 0.14; *p* = 0.0045; WT met-BDNF = 1.58 ± 0.15; *p* = 0.0008). On the other hand, expression of either val-BDNF or met-BDNF decreased postsynaptic GluA1 surface levels in *Mecp2* KO neurons (Figures 5A,C; KO val-BDNF = 0.97 ± 0.1; *p* = 0.0327; KO met-BDNF = 0.91 ± 0.17; *p* = 0.0494).

### Opposite to val-BDNF, met-BDNF Reduces mEPSC Frequency in Wildtype Neurons, in Addition to Reducing mEPSC Amplitudes in *Mecp2* Knockout and Wildtype Neurons

**Figure 6:**
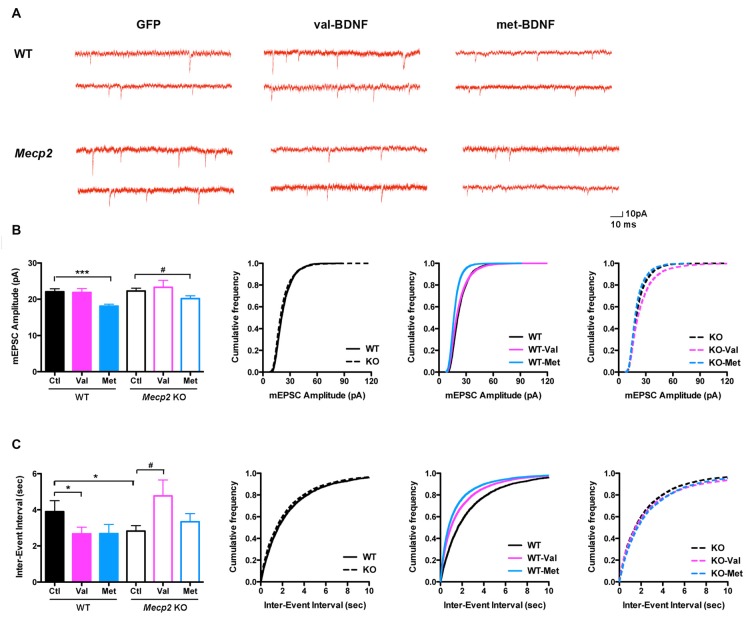
**(A)** Representative miniature excitatory postsynaptic currents (mEPSC) recordings from WT and *Mecp2* KO neurons expressing either soluble GFP alone, val-BDNF/soluble GFP, or met-BDNF/soluble GFP. **(B)** Average mEPSC amplitudes (for each cell) and cumulative probability distributions of all mEPSC amplitudes in WT and *Mecp2* KO neurons. **(C)** Average mEPSC inter-event intervals (for each cell) and cumulative probability distributions of all mEPSC inter-event intervals from WT and *Mecp*2 K*O* neurons. *p < 0.05, ***p < 0.001 compared to WT; ^#^*p* < 0.05 compared to *Mecp2* KO.

To characterize the functional role of *BDNF* SNP in synaptic function, we recorded mEPSC from pyramidal neurons in primary cultures from postnatal WT and *Mecp2* KO mice. The amplitude of mEPSCs was not significantly different between WT and *Mecp2* KO neurons (Figures 6A,B; WT Ctl = 22.07 ± 0.82 pA, *n* = 12 neurons; KO Ctl = 22.25 ± 0.176 pA, *n* = 17; *p* = 0.4393). On the other hand, the inter-event interval of mEPSCs was smaller in *Mecp2* KO neurons (Figures 6A,C; WT Ctl = 3.0 ± 0.6 s; KO Ctl = 2.82 ± 0.3 s; *p* = 0.0458), indicating a higher mEPSCs frequency. Since *Mecp2* KO neurons showed fewer excitatory synapses (Figure 5), this higher mEPSCs frequency might reflect differences in presynaptic transmitter release from individual preterminals.

Consistent with the effects of bath-applied recombinant mature BDNF protein (Tyler and Pozzo-Miller, 2001; Amaral and Pozzo-Miller, 2012), expression of val-BDNF increased mEPSC frequency in WT neurons (Figures 6A,C; WT val-BDNF = 2.67 ± 0.37 s; *n* = 17; *p* = 0.0382), without affecting mEPSC amplitude (Figures 6A,B; WT val-BDNF = 21.86 ± 1.08 pA; *p* = 0.4426). Intriguingly, val-BDNF expression decreased mEPSC frequency in *Mecp2* KO neurons (Figures 6A,C; KO val-BDNF = 4.77 ± 0.89 s; *n* = 12; *p* = 0.0289), without affecting mEPSC amplitude (KO val-BDNF = 23.29 ± 1.92 pA; *p* = 0.3131). On the other hand, expression of met-BDNF significantly decreased the amplitude of mEPSCs in both WT and *Mecp2* KO neurons (Figures 6A,B; WT met = 18.09 ± 0.52 pA, *n* = 13; *p* = 0.0002; *Mecp2* met = 20.16 ± 0.78 pA, *n* = 13; *p* = 0.0372), without affecting mEPSC frequency (Figures 6A,C; WT met-BDNF = 2.68 ± 0.51 s; *p* = 0.0666; KO met-BDNF = 3.34 ± 0.45 s; *p* = 0.1646).

## Discussion

The val66met SNP in the human *BDNF* gene is carried by approximately 30% of people worldwide and has been associated with cognitive deficits (Egan et al., 2003; Hariri et al., 2003; Harris et al., 2006; Liu et al., 2012). In this SNP, val at position 66 is changed to met in the pro region of proBDNF (Lu, 2003b; Hartmann et al., 2012). This SNP does not affect the expression levels or intracellular signaling triggered by the mature BDNF protein. However, the intracellular distribution and activity-dependent secretion are significantly impaired in neurons expressing met-BDNF, resulting in met-BDNF-GFP clustered in the perinuclear regions rather than in synaptic regions of dendrites and axons (Lu, 2003b; Hartmann et al., 2012). The inability of met-BDNF-GFP to be transported to neuronal processes and localized to synapses is thought to be due to impaired binding of met-BDNF to the sorting protein sortilin, which interacts with the pro region of BDNF and directs it from the trans-Golgi network into the regulated secretory pathway (Hartmann et al., 2012; Baj et al., 2013). The results presented here confirm that met-BDNF distribution is restricted to somata and only partially transported to the proximal area of primary dendrites in hippocampal neurons of both WT and *Mecp2* KO mice.

BDNF plays a critical role in activity-dependent dendritic and synaptic development (Chapleau et al., 2009b). Consisted with previous studies, our findings show that val-BDNF promotes dendritic growth and branching, and increases dendritic spine density and the volume of individual spines in hippocampal neurons. On the other hand, met-BDNF reduces dendritic length and branching, and fails to increase dendritic spine density. The function of BDNF relies on its proper trafficking to axons and dendrites, as well as sorting to the regulated secretory pathway, which allows Ca^2+^-dependent release. met-BDNF reduces the intracellular trafficking of BDNF messenger RNA (mRNA) to dendrites (Chiaruttini et al., 2009; Liu et al., 2012), and impairs the regulated BDNF secretion at synaptic sites (Egan et al., 2003; Chen et al., 2004), thus affects the dendritic growth. Therefore, met-BDNF hijacks val-BDNF, producing an overall deficit in BDNF trafficking into release-ready dense core vesicles. We found met-BDNF caused a decreased spine density, but not statistically different from control. Since we only analyzed spines from proximal (primary and secondary) dendrites, we cannot exclude that spines from distal dendrites may show larger differences, as Liu et al. (2012) reported in the prefrontal cortex.

Reduction in the size and complexity of dendritic arbors are common in disorders associated with intellectual disability, such as Rett syndrome (Kaufmann and Moser, 2000). Consistent with previous studies in autopsy brains from RTT individuals and symptomatic *Mecp2* KO mice (Armstrong et al., 1995; Fukuda et al., 2005; Schüle et al., 2008; Belichenko et al., 2009; Chapleau et al., 2009a), hippocampal neurons from newborn *Mecp2* KO mice maintained in primary culture have reduced dendritic complexity and lower spine density than WT neurons. These deficits are due to both a failure of the formation as well as of the maintenance of dendritic arbors (Baj et al., 2014). Expression of val-BDNF fully rescues dendritic growth and spine density in *Mecp2* KO neurons, as shown previously (Larimore et al., 2009). On the other hand, met-BDNF reduces dendritic complexity in *Mecp2* KO neurons, which correlates well with the observations of that smaller hippocampal volumes in humans and rodents carrying the met-BDNF allele (Egan et al., 2003; Hariri et al., 2003; Szeszko et al., 2005; Chen et al., 2006; Baj et al., 2013).

The number of bona fide dendritic spines (i.e., with a well-defined head) is a consistent estimate of excitatory synapses, while their volume is correlated with the strength of the particular synapse they receive (Bourne and Harris, 2008; Swanger et al., 2011). Even though *Mecp2* KO neurons have fewer excitatory spine synapses (identified by the co-localization of presynaptic VGLUT1 and postsynaptic GluA1), the volume of each individual spine is larger than in WT neurons. Fewer excitatory synapses may reflect delayed neuronal maturation, since adult newborn neurons in the dentate gyrus have lower dendritic spine density than their mature neighboring neurons (Smrt et al., 2007). However, the surface levels of synaptic GluA1 is higher in *Mecp2* KO neurons, consistent with stronger synapses that saturate long-term synaptic plasticity (Li et al., 2016), a consistent impairment of several mouse models of RTT (Chahrour and Zoghbi, 2007).

Consistent with numerous studies of the effects of bath-applied recombinant BDNF and expression of the rodent *Bdnf* gene, expression of the human val-BDNF increased dendritic spine density and the volume of individual dendritic spines in neurons from both WT and *Mecp2* KO mice, which provides support that BDNF-based therapies might be beneficial for RTT individuals. However, expression of met-BDNF failed to affect spine density in WT neurons, and in fact resulted in a reduction of dendritic spine volume in *Mecp2* KO neurons, which raises caution about the consequences of therapies aimed at increasing BDNF expression in individuals carrying the *BDNF* met allele, at least those that also harbor *MECP2* mutations.

The enhancement of glutamatergic synaptic transmission by BDNF is a consistent observation in hippocampal neurons (Lessman et al., 1994; Levine et al., 1998; Li et al., 1998). For example, the increase in mEPSC frequency in neurons expressing val-BDNF (without changes in mEPSC amplitude) is consistent with the presynaptic effects of bath-applied recombinant BDNF to CA1 pyramidal neurons (Tyler and Pozzo-Miller, 2001; Amaral and Pozzo-Miller, 2012). Previously, we described more frequent and larger mEPSCs in CA1 pyramidal neurons of adult symptomatic *Mecp2* KO mice compared to age-matched WT littermates, together with higher synaptic GluA1 levels and larger spine volumes, indicating stronger synaptic strength (Li et al., 2016). Here, we found that mEPSC amplitude is not affected in cultured pyramidal neurons from neonatal *Mecp2* KO mice. This apparent inconsistency could be due to the different developmental ages (neonatal vs. postnatal-day 60), or the expression of soluble GFP in cultured neurons, which was used for identification of transfected neurons in this study. Interestingly, the expression of val-BDNF decreased mEPSC frequency in *Mecp2* KO neurons, which would be beneficial by reducing the atypically enhanced synaptic strength in *Mecp2* KO neurons. On the other hand, met-BDNF decreased mEPSC amplitude in both WT and *Mecp2* KO neurons, suggesting that glutamatergic transmission is impaired. Consistently, the *BDNF* Val66Met polymorphism impairs NMDA receptor-dependent synaptic plasticity in various brain regions of *BDNF*^met/met^ mice (Ninan et al., 2010; Liu et al., 2012; Pattwell et al., 2012; Galvin et al., 2015; Jing et al., 2017), although some studies have described normal basal glutamatergic neurotransmission (Ninan et al., 2010; Pattwell et al., 2012) or increased (Jing et al., 2017).

In conclusion, expression of the human *BDNF* val-66-met SNP has deleterious consequences on dendritic complexity, the density and morphology of excitatory synapses on dendritic spines, as well as synaptic transmission in *Mecp2* KO neurons, suggesting that the met-BDNF variant contributes negatively to RTT pathophysiology. The outcome of therapies aimed at increasing *BDNF* expression may depend on the *BDNF* val-66-met SNP genotype, at least in those RTT individuals that also harbor *MECP2* mutations.

## Author Contributions

XX designed and performed experiments, analyzed data and wrote the manuscript; JG performed experiments and analyzed data; RE performed experiments and analyzed data; SN performed experiments and analyzed data; LP-M designed experiments, analyzed data and wrote the manuscript.

## Conflict of Interest Statement

The authors declare that the research was conducted in the absence of any commercial or financial relationships that could be construed as a potential conflict of interest.
